# Shikonin Protects PC12 Cells Against β-amyloid Peptide-Induced Cell Injury Through Antioxidant and Antiapoptotic Activities

**DOI:** 10.1038/s41598-017-18058-7

**Published:** 2018-01-08

**Authors:** Yuna Tong, Lan Bai, Rong Gong, Junlan Chuan, Xingmei Duan, Yuxuan Zhu

**Affiliations:** 1 Department of Nephrology, The Third People’s Hospital of Chengdu, Chengdu, 610031 China; 2 Department of Pharmacy, Sichuan Academy of Medical Science & Sichuan Provincial People’s Hospital, School of Medicine, University of Electronic Science and Technology of China, Chengdu, 610072 China

## Abstract

Excessive accumulation of β-amyloid (Aβ) is thought to be a major causative factor in the pathogenesis of Alzheimer’s disease (AD). Pretreating Aβ-induced neurotoxicity is a potential therapeutic approach to ameliorate the progression and development of AD. The present study aimed to investigate the neuroprotective effect of shikonin, a naphthoquinone pigment isolated from the roots of the traditional Chinese herb *Lithospermum erythrorhizon*, on Aβ_1–42_-treated neurotoxicity in PC12 cells. Pretreating cells with shikonin strongly improved cell viability, decreased the malondialdehyde and reactive oxygen species (ROS) content, and stabilized the mitochondrial membrane potential in Aβ_1–42_-induced PC12 cells. In addition, shikonin strongly improved the response of the antioxidant system to ROS by increasing the levels of superoxidedismutase, catalase and glutathione peroxidase. Furthermore, shikonin has the ability to reduce proapoptotic signaling by reducing the activity of caspase-3 and moderating the ratio of Bcl-2/Bax. These observations indicate that shikonin holds great potential for neuroprotection via inhibition of oxidative stress and cell apoptosis.

## Introduction

Alzheimer’s disease (AD) is the most common neurodegenerative disease, and it is typified by progressive brain degeneration and deterioration of cognitive function in the elderly people^1–3^. Due to a lack of effective treatments, AD has become one of the most devastating diseases in the world. The neuropathological characteristics of AD consist of deposition of extracellular senile plaques formed by excessive aggregation of β-amyloid protein (Aβ), neurofibrillary tangles, and neuron loss induced by neuronal apoptosis. Deposition of Aβ in the brain has been considered to be a critical effect in the progression of AD^1,4^. Aβ is the main component of senile plaques that has been implicated in kinds of physiological processes, such as cell survival and synaptic activity^5,6^. However, studies have suggested that changes in Aβ_1–42_ physicochemical properties and concentration potentially trigger its transition from physiological to pathological^7^.

The exact mechanisms of Aβ-induced neurotoxicity are still remain obscure, it has been reported that pathological deposition of Aβ leads to oxidative stress and a series of cascade reactions of apoptosis, inducing the progressive degeneration of cognition functions in AD patients. Thus, several researches have explored the activity of antioxidant and antiapoptotic drugs to ameliorate AD. Currently, the pharmacological treatment used to delay cognitive dysfunction in AD patients principally includes two types of drugs in the clinic, acetylcholinesterase inhibitors and glutamate modulators^8^. In addition, it has been reported that several alternative approaches have preventative effects on the progression of AD, including anti-Aβ antibodies, inhibitors of β- and γ-secretases, antioxidants and anti-inflammatory agents^9–12^. However, the stability, validity, cost, safety and development time restrict the use of these treatments for preventing AD^13^. Thus, searching for a safer and more effective drug for the treatment of AD remains an important challenge in drug discovery.

**Figure 1:**
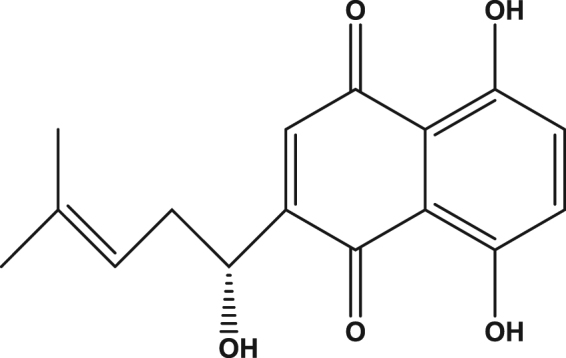
Chemical structure of shikonin.

Shikonin, a naphthoquinone pigment, is the chief active component extracted from the root of *Lithospermum erythrorhizon*, which has long been used in traditional oriental medicine for wound healing, urticaria and other allergic diseases (Fig. 1)^14^. It has been reported that shikonin possesses several pharmacological properties, such as antioxidant, anti-platelet activation, antiatherosclerosis, antithrombotic, anti-inflammatory, antitumor and antimicrobial properties^15–20^. These effects of shikonin are considered to be associated with its oxygen radical scavenging function^21,22^. According to reports, shikonin has the ability to protect against several types of reactive oxygen species (ROS)^22,23^, indicating that shikonin could protect against neurodegeneration from oxidative damage. Furthermore, it is suggested that shikonin plays a significant protective role in brain and hepatic ischemia/reperfusion injury by reducing ROS^22,24,25^. In this study, we examined the pro-survival activity of shikonin against oxidative damage induced by Aβ_1–42_ toxicity in PC12 cells. We determined the protective activity of shikonin on Aβ-induced damage and cell apoptosis in PC12 cells and completed initial research to investigate the underlying mechanism.

## Results

### Effect of Shikonin on Aβ_1–42_-Induced Cytotoxicity in PC12 cells

**Figure 2:**
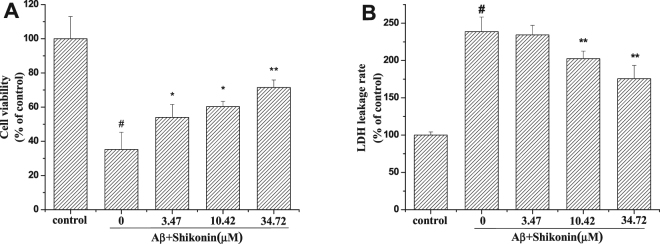
Protective effect of shikonin to against Aβ_1–42_-induced cytotoxicity in PC12 cells. Cell viability was measured with MTT (**A**) and LDH release (**B**) assays. Data are shown as the percent of values in control value, and the values are given as mean ± SD (n = 5). ^#^ *P < *0.01 compared with the control group (no Aβ_1−42_); *P < 0.05 and **P < 0.01 compared with the Aβ_1–42_-induced group.

We applied 100 µM Aβ_1–42_ aggregates to all PC12 cell groups throughout our experiments (Figs S1 and S2, Supporting Information). As indicated in 3-(4, 5-dimethyldiazol-2-yl)-2,5-diphenyltetrazolium bromide (MTT) assay of cell viability, treatment of PC12 cells with 100 µM Aβ_1–42_ for 12 h induced cytotoxicity, as demonstrated by the cell viability reduction to 35.27% compared with control group (Fig. 2A). When the cells were pretreated with serial concentrations of shikonin (3.47, 10.42, 34.72 µM) for 12 h, the cell viability was significantly increased (54.01, 60.29 and 71.51% of the control value, respectively) in a concentration-dependent manner. Furthermore, in MTT assay and flow cytometric detection of Aβ-induced SH-SY5Y cells, we found that viability of cells pretreated with shikonin was also strongly improved in a concentration-dependent manner (Figs S3, S4 and S5, Supporting Information).

As indicated in lactate dehydrogenase (LDH) release rate test of cell viability, treatment of PC12 cells with 100 µM Aβ_1–42_ for 12 h caused a 238.66% increase in LDH release rate compared to control group (Fig. 2B). When the cells were pretreated with serial concentrations of shikonin (3.47, 10.42, 34.72 µM) for 12 h, the LDH release rate was significantly decreased (234.35, 202.44 and 175.66% of the control value, respectively) in a concentration-dependent manner.

**Figure 3:**
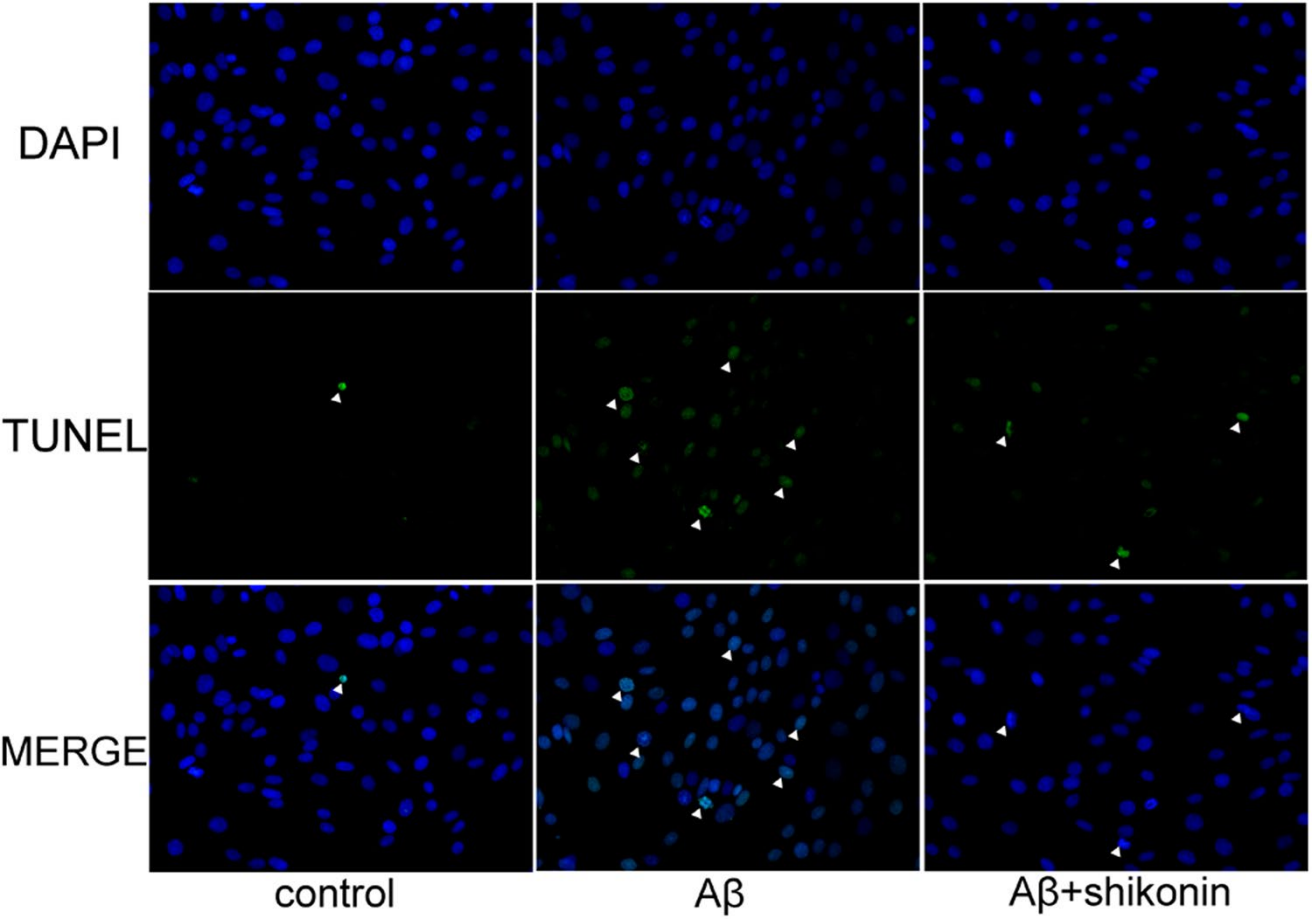
Protective effect of shikonin against Aβ_1–42_-induced apoptosis in PC12 cells analyzed with TUNEL staining (400×). Blue: PC12 cell nuclei counterstained with DAPI. Green: TUNEL staining of nuclei exhibiting DNA fragmentation. PC12 cells were pretreated with shikonin for 12 h, and then Aβ_1–42_ was added to the culture for 12 h. (**A**) control; (**B**) 100 µM Aβ_1–42_ treated alone; (**C**) 100 µM Aβ_1–42_ with 34.72 µM shikonin.

The effects of shikonin pretreatment on Aβ_1–42_-induced apoptosis in PC12 cells were detected using a TUNEL assay (Fig. 3). PC12 cells treated with Aβ_1–42_ showed obviously different staining activity than untreated cells, signifying apoptotic behavior. However, after pretreatment of PC12 cells with 34.72 µM shikonin, the Aβ_1–42_ resulted in significantly less staining, suggesting lower level of apoptosis. These results demonstrate that shikonin protects PC12 cells and SH-SY5Y cells from Aβ_1–42_-induced cytotoxicity and improves cell viability.

### Effect of Shikonin on Oxidative Stress in PC12 cells

**Figure 4:**
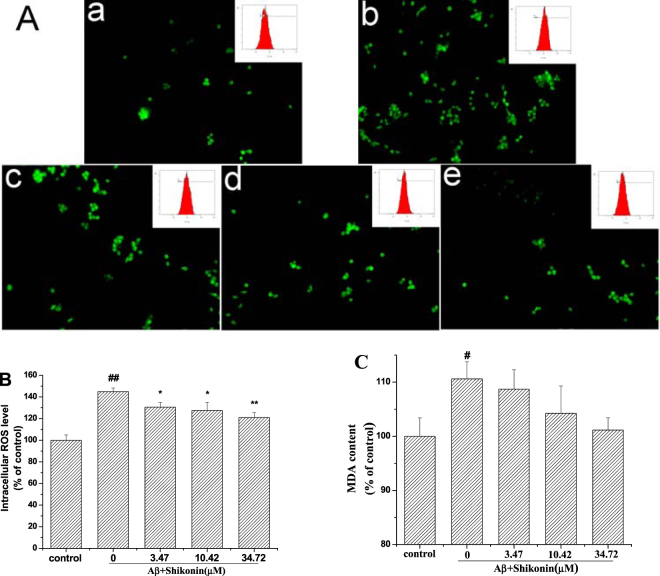
Effect of shikonin on Aβ_1–42_-induced oxidative stress in PC12 cells. Cells were induced with no Aβ_1−42_ (control) or with 100 μM Aβ_1−42_ following pretreatment with serial concentrations of shikonin. (**A**) ROS formation was detected with a fluorescence microscope (200×) and flow cytometry (insets): (a) control, (b) 100 µM Aβ_1–42_ treated alone, (c) 100 µM Aβ_1–42_ + 3.47 µM shikonin, (d) 100 µM Aβ_1–42_ + 10.42 µM shikonin, (e) 100 µM Aβ_1–42_ + 34.72 µM shikonin. (**B**) Detected fluorescence levels from the ROS assay are shown as the percentage of values in the untreated control group (mean ± SD; n = 3). (**C**) Results of MDA assay are shown as the percentage of values in the untreated control group (mean ± SD; n = 5). ^#^ *P* < 0.05 and ^##^ *P* < 0.01 compared with the control group (no Aβ_1−42_); *P < 0.05 and **P < 0.01 compared with the Aβ_1–42_-induced group.

Oxidative stress was detected by testing the level of intracellular ROS (Fig. 4A,B) and malondialdehyde (MDA) (Fig. 4C). After exposure to 100 µM Aβ_1–42_ for 12 h, ROS production and the MDA level were increased to 144.94% and 110.61% of the control value respectively. When the PC12 cells were pretreated with different concentrations of shikonin (3.47, 10.42, 34.72 µM) for 12 h, the intracellular ROS level (130.53, 127.51 and 120.95% of the control value, respectively) and the MDA level (108.71, 104.25 and 101.15% of the control value, respectively) were significantly decreased in a concentration-dependent manner.

### Effects of Shikonin on Aβ_1–42_- induced anti-oxidative enzymes in PC12 Cells

**Figure 5:**
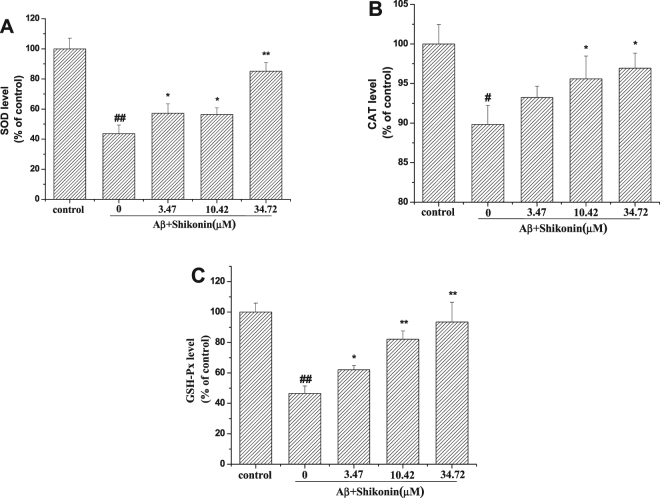
Effect of shikonin on the activity of anti-oxidative enzymes in PC12 cells. PC12 cells were induced with no Aβ_1−42_ (control) or with 100 μM Aβ_1−42_ following pretreatment with serial concentrations of shikonin. (**A**) Effect of shikonin on Aβ_1−42_-induced SOD levels. (**B**) Effect of shikonin on Aβ_1−42_-induced CAT levels. (**C**) Effect of shikonin on Aβ_1−42_-induced GSH-Px levels. Data are shown as the percentage of control group (mean ± SD; n = 5). ^#^ *P* < 0.05 and ^##^ *P* < 0.01 compared with the control group (no Aβ_1−42_); *P < 0.05 and **P < 0.01 compared with the Aβ_1–42_-induced group.

To determine whether the cell protection effect of shikonin is associated with the activity of anti-oxidative enzymes, the levels of superoxidedismutase (SOD) (Fig. 5A), catalase (CAT) (Fig. 5B) and glutathione peroxidase (GSH-Px) (Fig. 5C) were assessed. After exposure to 100 µM Aβ_1–42_ for 12 h, the levels of SOD, CAT and GSH-Px were decreased to 43.67%, 89.83% and 46.55% that of the control values respectively. When the PC12 cells were pretreated with different concentrations of shikonin (3.47, 10.42, 34.72 µM) for 12 h, the intracellular SOD level (57.24, 56.47 and 85.14% of the control value, respectively), CAT level (93.22, 95.61 and 96.95% of the control value, respectively) and GSH-Px level (62.07, 82.15 and 93.44% of the control value, respectively) were significantly ameliorated in a concentration-dependent manner.

### Effect of Shikonin on Aβ_1–42_-induced Mitochondrial Membrane Potential in PC12 cells

**Figure 6:**
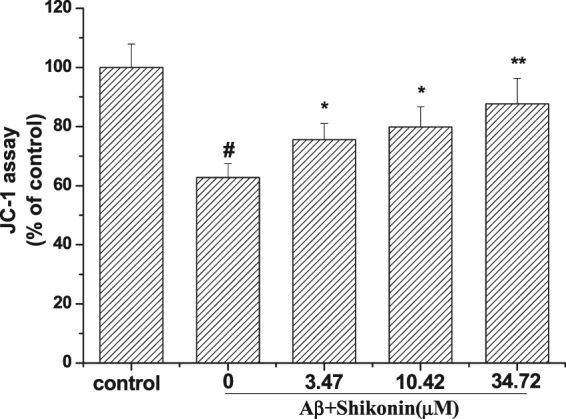
Effect of shikonin on changes in the mitochondrial membrane potential induced by Aβ_1–42_. PC12 cells were induced with no Aβ_1−42_ (control) or with 100 μM Aβ_1−42_ following pretreatment with serial concentrations of shikonin. The alteration of mitochondrial membrane potential was detected with JC-1 staining. Data are shown as the percentage of values in the untreated control group (mean ± SD; n = 3). ^#^ *P < *0.01 compared with the control group (no Aβ_1−42_); *P < 0.05 and **P < 0.01 compared with the Aβ_1–42_-induced group.

The mitochondrial membrane potential was assessed using a JC-1 assay (Fig. 6). After PC12 cells were treated with 100 µM Aβ_1–42_ for 12 h, the mitochondrial membrane potential was decreased to 62.75% compared with the control group. When PC12 cells were pretreated with serial concentrations of shikonin for 12 h, the mitochondrial membrane potential was significantly improved (75.53, 79.86 and 87.67% of the control value) in a concentration-dependent manner.

### Effects of Shikonin on Aβ_1–42_-induced Apoptotic Protein Expression and Activation in PC12 Cells

**Figure 7:**
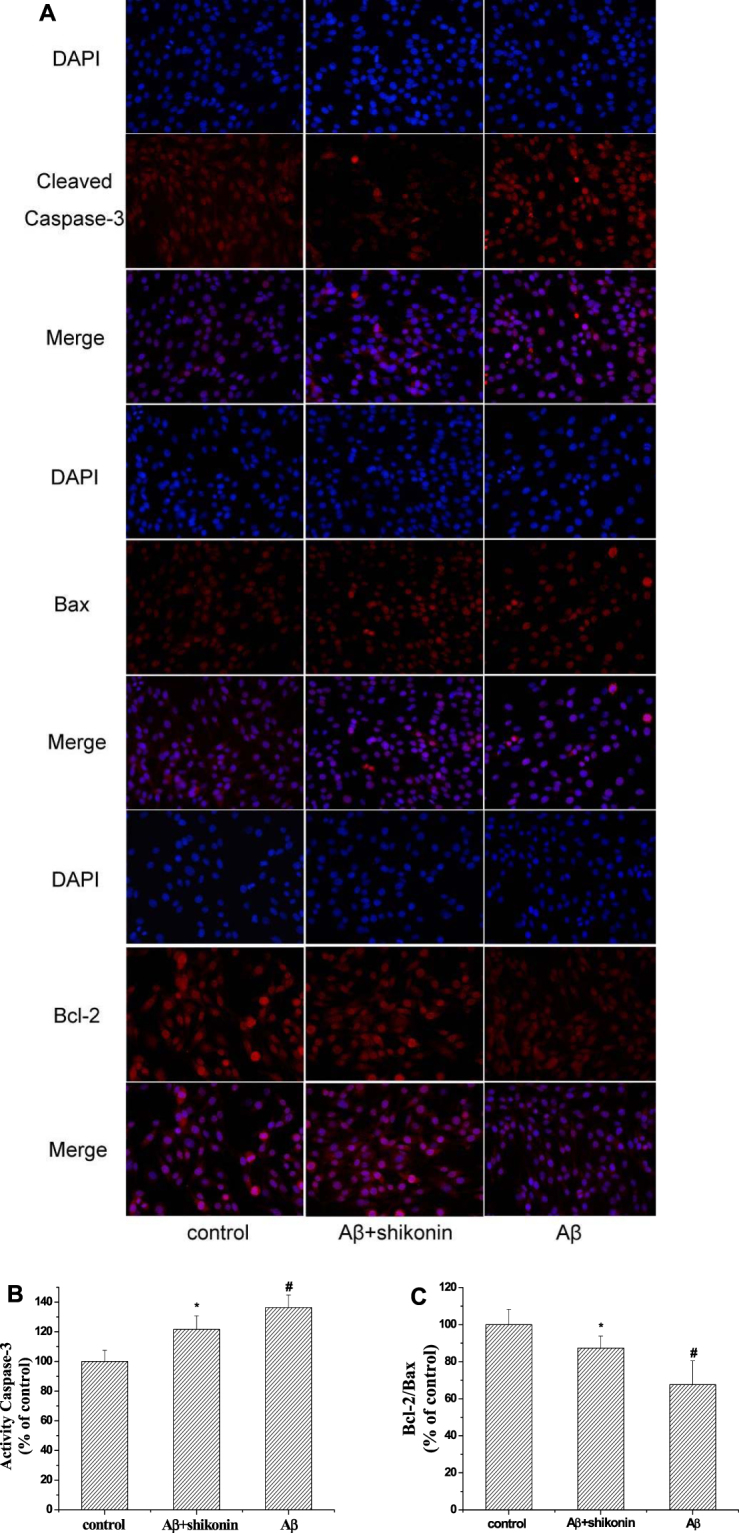
Inhibitory effect of shikonin on Aβ_1−42_-induced changes in activated caspase-3 and the Bcl-2/Bax ratio in PC12 cells. Cells were induced with no Aβ_1−42_ (control) or with 100 μM Aβ_1−42_ following pretreatment with shikonin. (**A**) Levels of Bax, Bcl-2 and cleaved caspase-3 were measured with a fluorescence microscope (400×). (**B**) Semiquantitative image analysis of cleaved caspase-3. Data are shown as the percentage of control group (mean ± SD; n = 3). (**C)** Semiquantitative image analysis of Bcl-2/Bax ratio. Data are shown as the percentage of control group (mean ± SD; n = 3). ^#^ *P < *0.01 compared with the control group (no Aβ_1−42_); *P < 0.05 compared with the Aβ_1–42_-induced group.

The ratio of antiapoptotic protein Bcl-2 to proapoptotic protein Bax and the relative levels of activation of caspase-3 have been reported to be correlated with apoptosis. To investigate the protective mechanisms of shikonin, the expression level of activated caspase-3 and the ratio of Bcl-2/Bax in PC12 cells were measured by immunofluorescence analysis (Fig. 7). Aβ_1–42_ strongly improved the content of cleaved caspase-3 to 136.33% compared with the control group and ameliorated the ratio of Bcl-2 to Bax to 67.63% compared with the control group. Shikonin at a concentration of 34.72 µM partially inhibited these changes in comparison to the Aβ_1–42_-induced group.

## Discussion

In this study, we have demonstrated that shikonin can partially protect PC12 cells from injury caused by Aβ-induced toxicity, oxidative stress and mitochondrial membrane depolarization. Although the precise mechanism of Aβ-induced neurotoxicity has not been completely elucidated, increasing evidence suggests that oxidative stress is closely involved in AD pathogenesis and ROS overproduction causes neuronal death in AD^26,27^. To assess the clinical potential of shikonin, we investigated its ability to ameliorate Aβ_1–42_-induced alterations in PC12 cells. The PC12 cell line has been widely employed as a general *in vitro* model to evaluate neuronal damage and neurotoxicity in AD. Furthermore, PC12 cells are able to provide high throughput and retain of a mature neuron phenotype^28^. The biochemistry and morphology of neuron growth factor (NGF)-induced PC12 cells are similar to neurons, and PC12 cells are particularly sensitive to Aβ peptides. In addition, several reports have suggested that Aβ_1–42_ not only leads to cytotoxicity and cell death but also induces ROS overproduction and mitochondrial dysfunction in PC12 cells^29^. We also observed these effects in our study. Therefore, the PC12 cells used in our experiments provide a reliable approach to determine whether shikonin affords protection against Aβ-induced cytotoxicity.

In our study, both MTT and LDH release tests were used to assess the neuroprotective effect of shikonin against the Aβ_1–42_-treated decrease in cell viability. PC12 cells apoptosis induced by Aβ_1–42_ was observed directly using TUNEL staining with and without shikonin pretreatment. Additionally, human neuroblastoma SH-SY5Y cells were used to further investigate the neuroprotective effect of shikonin against Aβ_1–42_-induced neurotoxicity. In the MTT assay, shikonin lowered Aβ_1–42_-induced SH-SY5Y cell death. Also the protective effects of shikonin noted in cell viability corresponded to the findings seen in cellular apoptotic. These experimental results indicate that shikonin has the ability to prevent PC12 and SH-SY5Y cells against the cytotoxicity induced by Aβ_1–42_. Further studies to investigate the mechanism of the neuroprotective activities indicated that shikonin pretreatment improved Aβ_1–42_-mediated oxidative stress, mitochondrial dysfunction and cellular apoptosis.

Oxidative stress is a disturbance in the balance between ROS production and the antioxidant defense system, which plays a major effect in the cell injury and neuronal degeneration in AD^30^. Several *in vitro* and *in vivo* studies have demonstrated that Aβ_1–42_ treatment causes ROS overproduction. Excessive ROS production is known to damage biomacromolecules in cells, consisting of proteins, lipids and DNA, eventually leading to neurodegeneration and depression^31,32^. In our study, we also found that Aβ_1–42_ treatment of PC12 cells not only increased ROS production but also increased the MDA content, and these results are in agreement with previous studies^27,33^. Furthermore, pretreating cells with shikonin attenuated these alterations in a concentration-dependent manner, indicating that the antioxidant effect of shikonin can mitigate apoptosis in AD.

Neurons depend on oxidative metabolism for survival. Under conditions of oxidative stress, there is an increase in ROS production and can result in cell damage or apoptosis. Under physiological conditions, there is a balance between the ROS production and the endogenous cellular antioxidant systems, including the cooperative action of SOD, CAT and GSH-Px^34–36^. It is known that SOD and GSH-Px serve as the primary line of intracellular defense against ROS by catalyzing their conversion to less reactive species. SOD is an important antioxidant enzyme in mitochondria against oxidative stress, and the activity of GSH-Px is to reduce the lipid hydroperoxides and free hydrogen to alcohols and water^34,35^. Additionally, CATs are commonly regarded as the second line of defense against dismutating peroxide into water and molecular oxygen^36^. When cells are treated with Aβ, sudden bursts of ROS cannot be eliminated by the cellular antioxidant enzymes, and the accumulation of ROS induces cellular membrane, protein and DNA oxidative damage. In our study, we discovered that Aβ_1–42_ significantly increased the levels of SOD, CAT and GSH-Px, indicating that these enzymes play a critical effect in ROS overproduction induced by oxidative stress. Pretreatment of PC12 cells with shikonin attenuated these Aβ_1–42_-treated alterations in a concentration-dependent manner, indicating that the protective activity of shikonin may be mediated by its antioxidant properties.

It is generally accepted that the mitochondrial membrane plays an important role in cell survival and death, especially under the influence of oxidative stress^27,37^. Mitochondrial dysfunction, leading to irreversible mitochondrial depolarization, has been found in Aβ-induced neurotoxicity in AD patients. ROS are produced in the mitochondria and then released into the cytoplasm, triggering oxidative stress and initiating cellular apoptosis. Our results illustrated that the mitochondrial membrane potential of PC12 cells treated with Aβ_1–42_ decreased, but this change was attenuated by shikonin. Therefore, according to our findings, we found that shikonin holds neuroprotective activity by inhibiting oxidative stress and apoptosis.

The Bcl-2 family plays an important factor in the mitochondrial apoptosis pathway induced by oxidative stress in PC12 cells exposed to Aβ_1–42_, and Bcl-2 family members consist of several pro-apoptotic including Bax and antiapoptotic including Bcl-2^38^. It has been reported that Bcl-2 has the ability to inhibit mitochondrial depolarization and ROS production, while Bax induces both of these processes^27^. Therefore, the ratio of Bcl-2 to Bax plays a pivotal role for cell survival and death. In addition, excessive ROS can induce the intermembrane protein to be released into the cytoplasm and eventually triggered caspase-3 activation, leading to cell apoptosis. Our present study indicated that Aβ_1–42_ ameliorated the Bcl-2/Bax expression ratio and improved the activation of caspase-3, consistent with previous researches in PC12 cells suggesting that these proteins play an important effect in mitochondria-mediated apoptosis induced by oxidative stress^39–41^. Our results suggested that pretreatment of shikonin in PC12 cells partially attenuated these Aβ_1–42_-treated alterations, indicating that the neuroprotective activities of shikonin may associate with its modulation on the expression of the Bcl-2 family.

In conclusion, our findings demonstrate that shikonin has a significant protective effect on Aβ_1–42_-induced neuronal injury. According to these *in vitro* studies, the protective effect of shikonin may be mediated by preventing oxidative stress and neuronal apoptosis. Additionally, further research is needed to verify and explore potential mechanisms underlying the findings.

## Materials and Methods

Shikonin, neuron growth factor (NGF), Dulbecco’s modified Eagle’s medium (DMEM), fetal bovine serum (FBS), horse serum (HS), and Aβ_1–42_ were obtained from Sigma–Aldrich Inc. (St Louis, MO, USA). The kits for malondialdehyde (MDA), superoxide dismutase (SOD), catalase (CAT), glutathione peroxidase (GSH-Px) and lactate dehydrogenase (LDH) were provided from Jiancheng Bioengineering Institute (Nanjing, Jiangsu, China). Reactive oxygen species (ROS) assay kit and mitochondrial membrance potential assay kit with JC-1 were obtained from Beyotime Institute of Biotechnology (Haimen, Jiangsu, China). Polyclonal antibodies against cleaved caspase-3, Bax and Bcl-2 were purchased from Cell Signaling Technology (Danvers, MA, USA).

### Cell culture

Undifferentiated rat pheochromocytoma PC12 cells were obtained from the American Type Culture Collection (Rockville, MD, USA) which were from passage 3 to 20. PC12 cells were cultured in high glucose DMEM containing 10% horse serum, 5% fetal bovine serum of penicillin-streptomycin and were maintained in a humidified atmosphere containing 5% CO_2_. PC12 cells were placed in poly D-lysine-coated plates and were treated with 5 ng/ml NGF to induce differentiation. The medium was changed every two days^38^.

### Cell Viability and LDH Assay

Cell viability was assessed by measuring the metabolism of 3-(4, 5-dimethyldiazol-2-yl)-2,5-diphenyltetrazolium bromide (MTT). Briefly, PC12 cells were seeded into 96-well plates with 8 × 10^3^ cells per well and incubated overnight for viability detection. After pretreatment with serial concentrations of shikonin (3.47, 10.42, 34.72 µM) for 12 h, the culture medium was replaced with medium containing 100 µM Aβ_1–42_ for 12 h. Then the cells were treated with 5 mg/ml MTT solution for 4 h at room temperature. The supernatants were then discarded, 150 µl of DMSO was added to solubilize the formazan crystals with shaking for 5 min. The absorbance at 570 nm of solution was detected using a in a microplate reader (Thermo, Varioskan Flash).

The activity of released LDH is an *in vitro* marker for cell injury. The extracellular and intracellular LDH content were assessed with a commercial assay kit. Briefly, PC12 cells were plated in 6-multiwell plates at 4 × 10^5^ cells/well for LDH activity determination. The cells were pretreated with serial concentrations of shikonin (3.47, 10.42, 34.72 µM) for 12 h, and then, the PC12 cells were insulted with 100 µM Aβ_1–42_ for 12 h. The culture medium was collected for the extracellular LDH activity assay. After washing three times in cold PBS, the adherent cells were collected by scraping and homogenized for the intracellular LDH activity assay. The absorbance of each sample was measured at 450 nm via microplate reader. LDH release was calculated as follows:

$${\rm{LDH}}\,{\rm{release}}\,{\rm{rate}}\,( \% )=\frac{{\rm{LDH}}\,{\rm{activity}}\,{\rm{in}}\,{\rm{the}}\,{\rm{culture}}\,{\rm{medium}}}{{\rm{LDH}}\,{\rm{activity}}\,{\rm{in}}\,{\rm{the}}\,{\rm{culture}}\,{\rm{medium}}\,+\,{\rm{LDH}}\,{\rm{activity}}\,{\rm{in}}\,{\rm{cells}}}\times 100 \% $$

### TUNEL staining

The degree of DNA fragmentation was determined by a terminal deoxynucleotidyl transferase nickend labeling (TUNEL) assay. In brief, PC12 cells were cultured on coverslips. After incubation with shikonin (34.72 µM) for 12 h and exposure with 100 µM Aβ_1–42_ for 12 h, media was aspirated and plates were rinsed with cold PBS buffer. The cells were fixed with 4% paraformaldehyde at 4 °C for 10 min. The cells were further incubated in blocking and treated with 0.1% TritonX-100 in 0.1% sodium acetate for 5 min. Thereafter, the cells were labeled by incubation with the TUNEL reagent and fluorescent dUTP mixture for 1 h at 37 °C. Nuclei were counterstained with 4′,6′-diamidino-2-phenylindole (DAPI). Subsequently, the cells were observed with a fluorescence microscope (Carl Zeiss Shanghai Co., Ltd).

### Lipid Peroxidation Assay

The malondialdehyde (MDA) content, a marker of lipid peroxidation, was tested by biochemical methods according to the instructions of reagent kits. Briefly, 4 × 10^5^ cells per well were cultured into 6-well plates. After pretreatment with serial concentrations of shikonin (3.47, 10.42, 34.72 µM) for 12 h, the cells were insulted with 100 µM Aβ_1–42_ for 12 h. At the end of the treatment, PC12 cells were rinsed with cold phosphate-buffered saline and homogenized in 0.5 ml of buffer solution (0.1 M, containing 0.05 mM EDTA). The homogenate was centrifuged at 4000 g for 10 min. The supernatant was collected for the MDA content assay.

### Dichlorofluorescein assay for ROS

The ROS level was monitored using a fluorescent probe 2’, 7’-dichlorodihydrofluorescin diacetate (DCFH-DA). Intracellular ROS oxidize nonfluorescent compound DCFH-DA to highly fluorescent compound dichlorofluorescein (DCF), which can be detected with a fluorescence microscope and flow cytometry. In brief, 4 × 10^5^ cells per well were cultured into 6-well plates. After pretreatment with serial concentrations of shikonin (3.47, 10.42, 34.72 µM) for 12 h, then the cells were insulted with 100 µM Aβ_1–42_ for 12 h. At the end of treatment, PC12 cells were rinsed with cold phosphate-buffered saline and treated with 10 µM DCFH-DA for 30 min at room temperature in a humid and dark environment. At the end of the incubation, cold phosphate-buffered saline was used to wash away the extracellular DCFH-DA molecules. Finally, the fluorescence of DCF was detected with a fluorescence microscope (Carl Zeiss Shanghai Co., Ltd.) and flow cytometry (Beckman Coulter).

### Antioxidant Systems Assay

The cultured cells were seeded into 6-well plates at 4 × 10^5^ cells per well. After pretreatment with serial concentrations of shikonin (3.47, 10.42, 34.72 µM) for 12 h, the cells were insulted with 100 µM Aβ_1–42_ for 12 h. At the end of the treatment, PC12 cells were rinsed with cold phosphate-buffered saline and homogenized in 0.5 ml of buffer solution. The homogenates were centrifuged, and the supernatants were used for the assay.

SOD, CAT and GSH-Px activities in PC12 cells were measured by means of kit assays according to the instructions of the manufacturers. The SOD activity was determined using the xanthine oxidase method at 550 nm wavelength with a spectrophotometer. The CAT activity was detected by biochemical method according to the instruction of reagent kit, and the absorbance of the test solution was measured at 405 nm wavelength with a spectrophotometer. The GSH-Px activity was assayed by quantifying the rate of oxidation of reduced glutathione to oxidized glutathione by H_2_O_2_ at 412 nm wavelength.

### Measurement of Mitochondrial Membrane Potential

The membrane-permeant JC-1 dye was used to monitor mitochondrial membrane potential of Aβ_1–42_-induced PC12 cells. 4 × 10^5^ cells per well were cultured into 6-well plates. After pretreatment with serial concentrations of shikonin (3.47, 10.42, 34.72 µM) for 12 h, the cells were insulted with 100 µM Aβ_1–42_ for 12 h. At the end of the treatment, PC12 cells were treated with JC-1 dye for 30 min. After incubation, the cells were washed three times with phosphate-buffered saline. The fluorescence was measured by flow cytometry (Beckman Coulter), depolarization in mitochondrial membrane potential of PC12 cells was quantified as the ratio of the red to green signal.

### Immunofluorescence Assay

Immunofluorescence assay was used to evaluate the levels of Bax, Bcl-2, and activated caspase-3 protein. Briefly, PC12 cells were cultured onto coverslips after incubation with shikonin (34.72 µM) for 12 h and exposured to 100 µM Aβ_1–42_ for 12 h. The medium was aspirated from the plates, and the cells were rinsed with phosphate-buffered saline. At the end of the treatment, PC12 cells were fixed for 10 min with 4% paraformaldehyde at 37 °C and rinsed with phosphate-buffered saline. Fixed cells were incubated with primary antibodies diluted in PBS (anti-cleaved caspase-3, 1:1000; anti-Bax, 1:1000; anti-Bcl-2, 1:500) overnight. The coverslips were washed and treated with Cy3-conjugated secondary antibody diluted 1:500 in PBS for 4 h. Nuclei were counterstained with 4′,6′-diamidino-2-phenylindole (DAPI). Subsequently, the cells were observed with a fluorescence microscope (Carl Zeiss Shanghai Co., Ltd.).

### Data analysis

Results are shown as the mean ± SD. Differences between two experimental conditions were evaluated with one-way ANOVA followed by Dunnett’s post-hoc test (version 17.0 software, SPSS Inc.). Differences were accepted statistically significant for *P* < 0.05.

## Electronic supplementary material

supporting information
