# Supplementary material for: Shikonin Protects PC12 Cells Against β-amyloid Peptide-Induced Cell Injury Through Antioxidant and Antiapoptotic Activities
Source: Sci Rep. 2018 Jan 8;8:26. doi: 10.1038/s41598-017-18058-7 (PMC5758797; doi:10.1038/s41598-017-18058-7)
Supplement: Supplementary file 1 — supporting information [file 41598_2017_18058_MOESM1_ESM.pdf]

Supporting Information for:

Shikonin Protects PC12 Cells Against β-amyloid Peptide-Induced Cell Injury Through Antioxidant and Antiapoptotic Activities

Yuna Tong, Lan Bai, Rong Gong, Junlan Chuan, Xingmei Duan, Yuxuan Zhu*

Experimental Section

Preparation and observation of Aβ$_{1–42}$ Aggregates.

Aβ$_{1-42}$ was dissolved in deionized distilled water at a concentration of 1 mM and incubated at 37°C for 7 d to form aggregated amyloid. After aggregation, the peptide was analyzed by transmission electron microscopy (TecnaiG2 F-20, FEI, Holland). Aβ$_{1–42}$ samples were applied onto glow-discharged copper grids and stained with 1% uranyl acetate.

Aβ$_{1–42}$ Treatment.

Cell viability was assessed by quantitative colorimetric assay via MTT method as described previously. Briefly, the PC12 cells induced differentiation by NGF were plated in 96-well plates at a density of 8 × 10^3 cells/well and incubated for 24 h for viability determination. The cells were treated with prepared Aβ$_{1-42}$ of different concentration (1, 5, 10, 20, 50, 100, 200 and 500 µM) for 12 h. Then MTT solution (5 mg/ml) was added to each well and incubated for 4 h at 37 ºC. The supernatants were then discarded, 150 µl of DMSO was added to solubilize the formazan crystals with shaking for 5 minutes. Cell viability was measured by reading absorbance at 570 nm in a plate reader (Thermo, Varioskan Flash).

Cell Culture.

SH-SY5Y (human neuroblastoma cells) were obtained from the American Type Culture Collection (Rockville, MD, U.S.A.) which are from passage 3 to 20. Cells were cultured in DMEM with high glucose (Hyclone, U.S.A.) supplemented with 10% FBS (Hyclone, U.S.A.), 100 IU/mL penicillin, and 100 µg/mL streptomycin. Cells were maintained at 37 °C in a humidified atmosphere containing 5% CO_2, and the culture medium was changed every other day.

Cell Viability Assay

Cell viability was measured using MTT reduction assay as described previously. Briefly, the SH-SY5Y cells were seeded on 96-well plates at a density of 4 × 10^4 cells/well and incubated for 24 h for viability determination. The cells were treated with prepared Aβ$_{1-42}$ of different concentration (1, 5, 10, 30, 50, 100, 200 µM) for 24 h. Then MTT solution (5 mg/ml) was added to each well and incubated for 4 h at 37 ºC. The supernatants were then discarded, 150 µl of DMSO was added to solubilize the formazan crystals with shaking for 5 minutes. Cell viability was measured by reading absorbance at 570 nm in a plate reader (Thermo, Varioskan Flash).

SH-SY5Y cells were seeded in 96-well culture plates (4 × 10^4 cells/well) for

viability assays. The cells were pre-incubated with shikonin at different concentrations (3.47, 10.42, 34.72 μM) for 12 h, then the culture medium was replaced with medium containing 30 μM $A\beta_{1-42}$ for 12 h. MTT solution (5 mg/ml) was added to each well and incubated for 4 h at 37□. The supernatants were then discarded, 150 μl of DMSO was added to solubilize the formazan crystals with shaking for 5 min. Cell viability was measured by reading absorbance at 570 nm in a plate reader (Thermo, Varioskan Flash).

Cell Apoptosis Assay

The quantitative analysis of apoptosis induced by different treatment groups was performed by Annexin V-FITC/PI double staining using the Cell Apoptotic analysis kit (Beyotime, Jiangsu, China). Briefly, SH-SY5Y cells were seeded on 6-well culture plates (5×10^5 cells/well) for cell apoptosis tests. At the end of the treatment, cells were harvested, washed with cold PBS, suspended in 0.5 ml binding buffer and stained by 5 μl Annexin V-FITC and 5 μl PI. The cells were incubated in the dark for 15 min and measured by Cytomics FC500 flow cytometer (Beckman Coulter, U.S.A.).

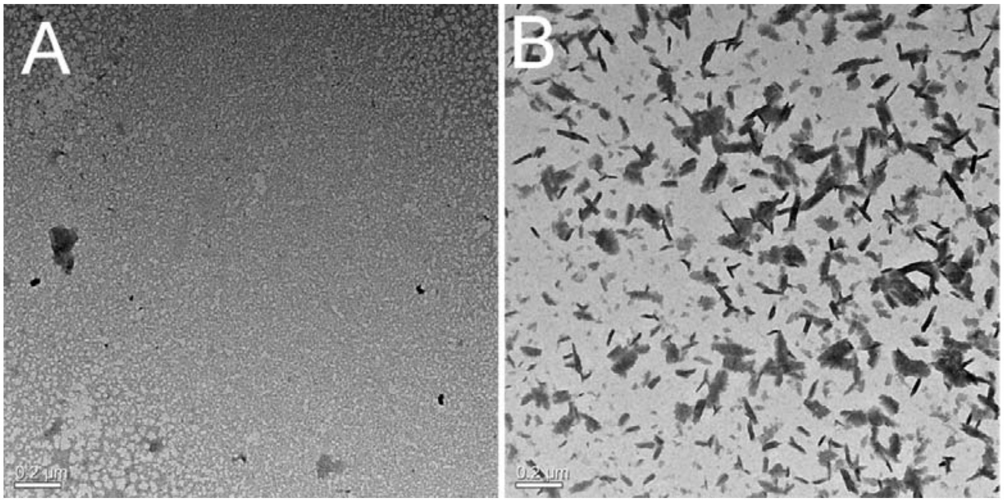

Figure S1. $A\beta_{1\text{-}42}$ aggregates formation observed by TEM. (A) $A\beta_{1\text{-}42}$ without treatment; (B) $A\beta_{1\text{-}42}$ incubated for 7 days at 37 ºC.

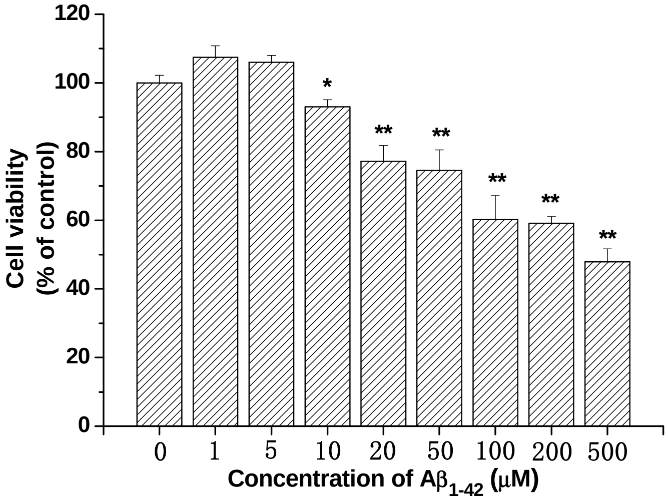

Figure S2. The PC12 cell was induced by treating with different concentrations of $A\beta_{1\text{-}42}$ for 12 h. Cell viability was measured using assays based on MTT. Data are expressed as percent of values in control group (no $A\beta_{1-42}$), and the values are given as mean ± SD (n= 5). *$P < 0.05$ and **$P < 0.01$ compared with the control group (no $A\beta_{1-42}$).

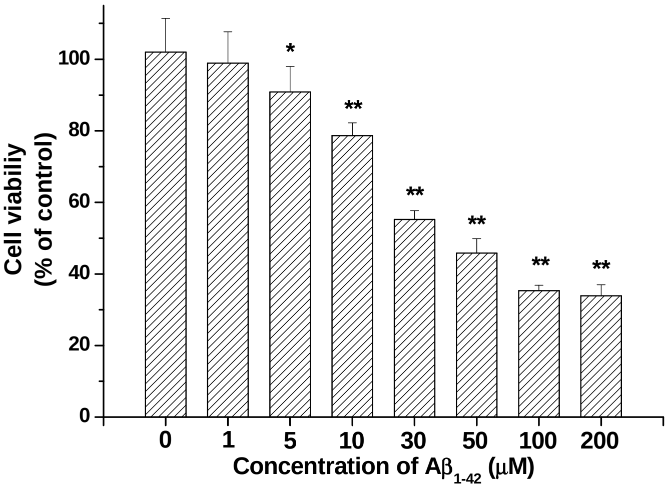

Figure S3. The SH-SY5Y cell was induced by treating with different concentrations of $A\beta_{1\text{-}42}$ for 12 h. Cell viability was measured using assays based on MTT. Data are expressed as percent of values in control group (no $A\beta_{1-42}$), and the values are given as mean ± SD (n= 5). *P < 0.05 and **P < 0.01 compared with the control group (no $A\beta_{1-42}$).

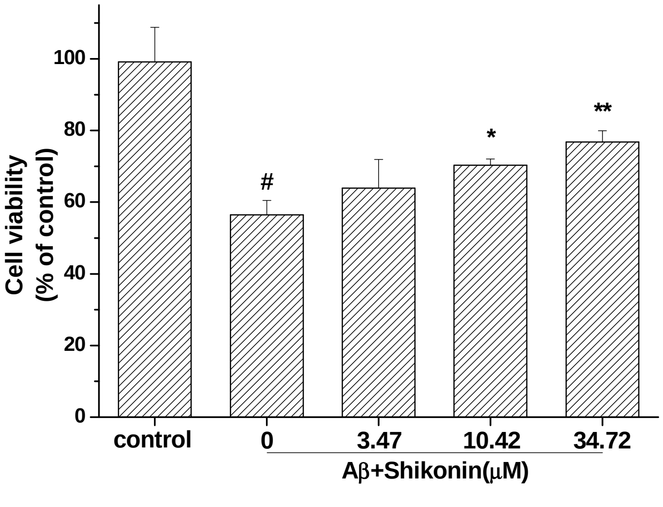

Figure S4. Protective effect of shikonin to against $A\beta_{1\text{-}42}$-induced cytotoxicity in SH-SY5Y cells. Cell viability was measured using assays based on MTT. Data are expressed as the percent of values in control group, and the values are given as mean ± SD (n = 5). $^{\#}P < 0.01$ compared with the control group (no $A\beta_{1-42}$); $^{*}P < 0.05$ and $^{**}P < 0.01$ compared with the $A\beta_{1\text{-}42}$-induced group.

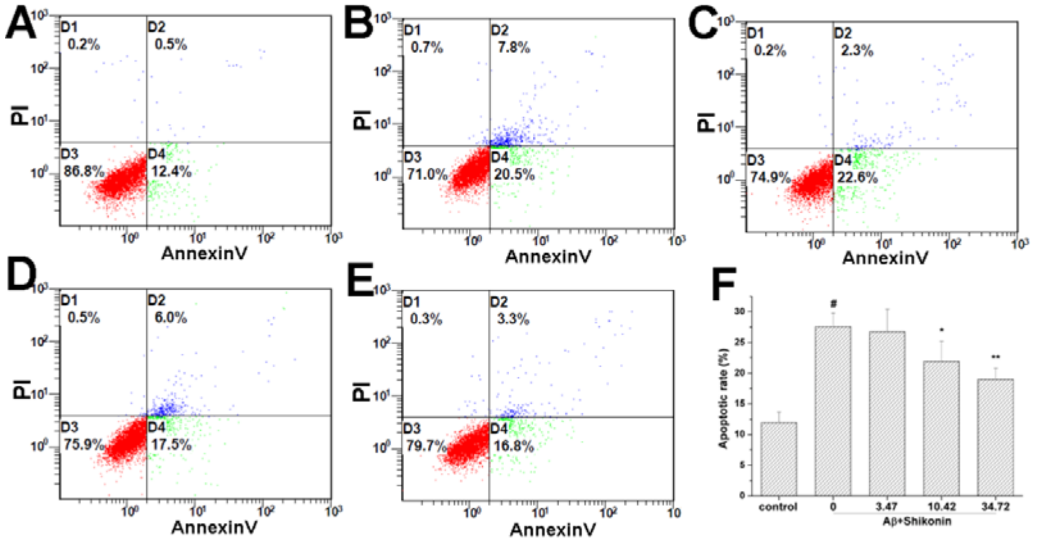

Figure S5. Effect of shikonin on A$\beta_{1\text{-}42}$-induced SH-SY5Y cell apoptosis. Apoptosis of SH-SY5Y cells was quantified with flow cytometry by combined staining with Annexin V/PI. (A) control, (B) 30 μM A$\beta_{1\text{-}42}$ treated alone, (C) 30 μM A$\beta_{1\text{-}42}$ + 3.47 μM shikonin, (D) 30 μM A$\beta_{1\text{-}42}$ + 10.42 μM shikonin, (E) 30 μM A$\beta_{1\text{-}42}$ + 34.72 μM shikonin, (F) Results are expressed as mean ± SD; n = 3. $^{\#}P < 0.01$ compared with the control group (no Aβ_{1-42}); *$P < 0.05$ and **$P < 0.01$ compared with the A$\beta_{1\text{-}42}$-induced group.
